# A Comparative Analysis of Lung Cancer Incidence and Tobacco Consumption in Canada, Norway and Sweden: A Population-Based Study

**DOI:** 10.3390/ijerph20206930

**Published:** 2023-10-17

**Authors:** Jing Chen

**Affiliations:** Radiation Protection Bureau, Health Canada, 775 Brookfield Road, Ottawa, ON K1A 1C1, Canada; jing.chen@hc-sc.gc.ca

**Keywords:** tobacco, smoking, lung cancer

## Abstract

Risk factors for developing lung cancer include tobacco smoking, exposure to second-hand smoke, radon gas, asbestos and other carcinogens, as well as air pollution and aging. Canada, Norway and Sweden are three Arctic countries that are very similar in socioeconomic status, similar in the social determinants of health and many environmental factors, including current smoking rates. However, lung cancer incidence rates differ significantly among the three countries. Since tobacco smoking is the principal risk factor for developing lung cancer, and there is a long lag time between tobacco consumption and lung cancer incidence at the population average level, a comparative analysis is conducted with historical data available publicly in the three countries to demonstrate how tobacco consumption in the past impacts lung cancer incidence decades late. The analysis shows that lung cancer incidence rates increase proportionally with the increasing tobacco consumption rates with a lag time of 20–30 years. On average, one more cigarette smoked per person per day can increase lung cancer cases by 2 to 5 per 100,000 population.

## 1. Introduction

Lung cancer risk factors include tobacco smoking, exposure to second-hand smoke, exposure to radioactive radon gas, exposure to asbestos and other carcinogens, as well as air pollution and aging. Research has shown that the majority of lung cancer cases—about 85%—are directly related to tobacco smoking, particularly cigarettes (https://www.lungcancercanada.ca/en-CA/Lung-Cancer/Causes/Smoking.aspx, accessed on 1 July 2023). For several decades, lung cancer has been the leading cause of cancer death, responsible for more cancer deaths among Canadians than the other three major cancer types (colorectal, breast and prostate) combined [1].

Canada, Norway and Sweden are three Arctic countries that are very similar with respect to socioeconomic status, long and healthy life and many environmental factors. The life expectancy for people in Canada, Norway and Sweden is very similar (80.4—81.1 years for men and 84.0–84.1 years for women) [2]. It is well known that air pollution is a risk factor for lung cancer. As indicated by the concentrations of fine particulate matter (PM2.5), ambient (outdoor) air quality is similar for the three Arctic countries: 6.39 in Canada, 6.30 in Norway and 5.96 in Sweden [3] (2019 data). In addition, there is little difference in key indicators of indoor air quality: for example, the proportion of the population with primary reliance on clean fuels and technologies for cooking is 100% for all three countries [4]. The population-weighted arithmetic mean indoor radon concentrations are 82 Bq/m^3^ in Canada [5], 89 Bq/m^3^ in Norway and 83 Bq/m^3^ in Sweden [6]. Finally, in 2019, adult daily tobacco smokers are reported to be 8.6% in Canada, 9.0% in Norway and 10.2% in Sweden [7].

There are studies that have shown that differences in lung cancer incidence between countries can be attributed to differences in lung cancer detection rates, and wealthier countries with greater access to healthcare and higher socioeconomic levels experience much higher rates of cancer incidence than poor countries [8,9,10]. A study on temporal trends of lung cancer incidence in 38 countries/regions has found that age-standardized incidence rates are positively correlated with the Health Development Index (HDI) [11]. The HDI is a summary measure of average achievement in key dimensions of human development: a long and healthy life, being knowledgeable and having a decent standard of living [12]. The health dimension is assessed via life expectancy at birth. The education dimension is measured using the mean of years of schooling for adults aged 25 years and more and expected years of schooling for children of school-entering age. The standard of living dimension is measured using the gross national income per capita. The HDI is a composite index using geometric mean from the scores of the three HDI dimension indices. According to the United Nations Development Programme [12], Canada, Norway and Sweden are among the top 15 countries with high HDI in the past decades. In 1990, the HDI was 0.86 for Canada, similar to 0.84 for Norway and 0.81 for Sweden. The HDI has improved year by year in the three countries. By 2020, HDI is 0.931 for Canada, also very similar but very slightly lower than 0.959 for Norway and 0.942 for Sweden.

With the above-mentioned similarities in social determinants of health, education, income level and environmental factors, it is hard for the general public to understand why Canadian lung cancer incidence rates are significantly higher than the rates in Norway and Sweden. For example, the reported age-standardized lung cancer incidence rate for Canadian women was 31.6 per 100,000 persons in 2012, significantly higher than 26.7 in Norway and 18.3 in Sweden [13].

At the population average level, there is a long lag time between tobacco smoking and the induction of lung cancer. Countries having similar smoking rates nowadays may have significantly different lung cancer incidence rates depending on the past smoking prevalence. To demonstrate the impact of past tobacco consumption on the current lung cancer incidence rate, a comparative analysis was conducted on the correlations between lung cancer incidence and tobacco consumption in three countries (Canada, Norway and Sweden) with very similar socioeconomic status and similar social determinants of health and environmental factors.

## 2. Materials and Methods

This comparative study uses publicly available historical data on lung cancer incidence rates and tobacco consumption in Canada, Norway and Sweden.

### 2.1. Lung Cancer Incidence

Lung cancer refers to cancer of the trachea, bronchus and lung (ICD 162). Lung cancer incidence rates are taken from the International Agency for Research on Cancer (IARC) cancer database [13] for Norway and Sweden from 1960 to 2020 and for Canada from 1983 to 2020. The data for 2020 are estimated values. Canadian data from 1969 to 1982 are from Canadian Cancer Statistics 1997 [14]. Lung cancer incidence rates (LCIR) are expressed in age-standardized incidence rates (ASIR) per 100,000 population. The long-term trends of LCIRs are needed for estimation of possible lag time between tobacco consumption and lung cancer incidence in a country.

### 2.2. Tobacco Consumption

Studies have shown that number of cigarettes smoked per person per day is better related to lung cancer incidence than percentage of smokers in a population [15,16,17,18]. Historical trends of tobacco consumption in units of number of cigarettes per person per day (cigarettes/person/day) are available in the book “International Smoking Statistics”, web edition [19].

### 2.3. Correlation Coefficient and Lag Time

The possible lag time between tobacco consumption and lung cancer incidence is examined in detail at the national level with tobacco consumption data from the 1950s to 2000s and lung cancer incidence rates from the 1960s to 2020. A lag time close to zero would be unrealistic, as would a lag time in excess of 40 years. Correlation coefficients between tobacco consumption (TC) in units of cigarettes per person per day and lung cancer incidence rate (LCIR) in age-standardized incidence rates (ASIR) per 100,000 population were calculated for lag times between 5 to 40 years (where data are available) using

(1) $$r=\frac{\sum_{n}\left(TC-{TC}_{avg})(ASIR-{ASIR}_{avg}\right)}{\sqrt{\sum_{n}{\left(TC-{TC}_{avg}\right)}^{2}}\sqrt{\sum_{n}{\left(ASIR-{ASIR}_{avg}\right)}^{2}}}$$

where ${TC}_{avg}$ and ${ASIR}_{avg}$ correspond to the mean *TC*s and *ASIR*s. The lag time is determined using the highest correlation coefficient.

### 2.4. Using Average Values to Reduce Uncertainties

Tobacco consumption surveys are normally conducted with a randomly selected portion of the population using questionnaires via telephone interviews. Sample sizes vary between surveys and over time. For example, there were 45 nationally representative surveys carried out in Canada between 1951 and 2011. Sample sizes varied from 1429 in the 1963 Gallup survey to 66,288 in the 1974 Canadian Smoking Habits Surveys [20]. In addition to the statistical uncertainties, tobacco products, the makeup of cigarettes and the composition of cigarette smoke have gradually changed over the past several decades [21]. Thus, reported annual tobacco consumption rates have large uncertainties.

Statistical uncertainties also exist in reported lung cancer incidence rates (variations in diagnostic practices and data collection) but to much lower degree than the uncertainties with tobacco consumption surveys.

Long lag time between tobacco smoking and lung cancer incidence indicates a chronic long-term effect of tobacco smoking on the induction of lung cancer. To consider the long-term effect of tobacco smoking and to limit the impact from various statistical uncertainties and fluctuations, 15-year averaged tobacco consumption rates (TC15) and 5-year averaged lung cancer incidence rates (LCIR5) are considered for the examination of their correlations.

## 3. Results

**Figure 1:**
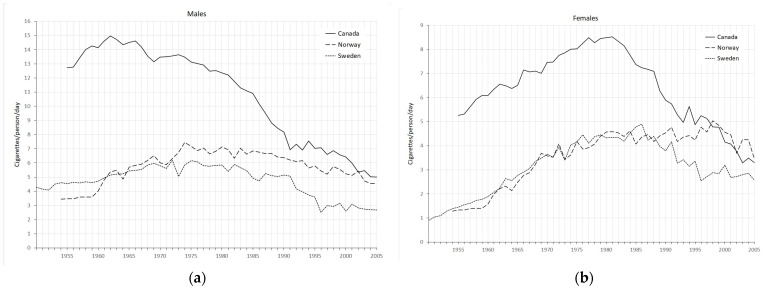
Trends of tobacco consumption rate for adults aged 15 and older: (**a**) males and (**b**) females.

Tobacco consumption data up to the year 2005 are presented in Figure 1. Before the 1990s, many more Canadians (both men and women) smoked than people living in Norway and Sweden. Tobacco consumption between Norwegians and Swedes was comparable and much lower.

**Figure 2:**
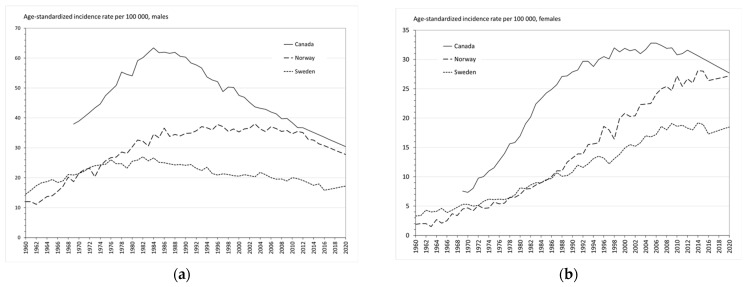
Trends of age-standardized lung cancer incidence rates per 100,000: (**a**) males and (**b**) females.

The age-standardized incidence rate is a weighted average of the age-specific incidence rates per 100,000 persons, where the weights are the proportions of persons in the corresponding age groups of the World Health Organization’s standard population [12]. The data are summarized in Figure 2. Lung cancer incidence trends in Canada, Norway and Sweden reflected the past differences in smoking prevalence of the three countries, as clearly demonstrated in Figure 2. The more people that had smoked, the higher the lung cancer incidence rate.

**Figure 3:**
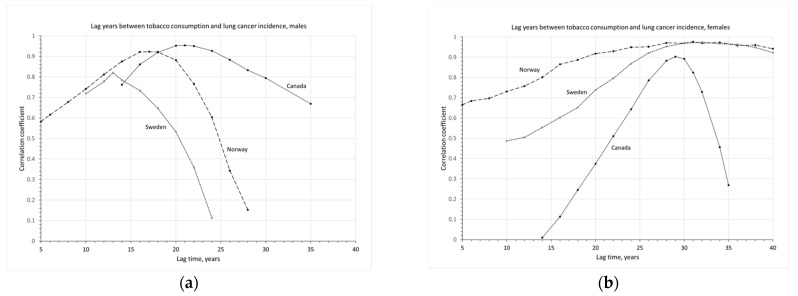
Correlation coefficient between tobacco consumption and lung cancer incidence as a function of lag time in years: (**a**) males and (**b**) females.

To quantitatively analyze the impact of tobacco consumption on the incidence of lung cancer, lag times between smoking and lung cancer incidence are determined for men and women, respectively. To determine the lag time, correlation coefficients between tobacco consumption and lung cancer incidence are calculated. In Canada, *LCIRs* were only available from 1969 onwards and *TC* from 1955 onwards. Therefore, the correlation coefficients for Canada are calculated for lag times between 14 to 35 years. The results are given in Figure 3.

For Canadians, the highest correlation of 0.95 is at a lag time of 21 years for men and 29 years for women, with the highest correlation of 0.90. There is an 8-year difference in lag time between Canadian men and women.

In Norway, the LCIRs for males were stabilized at 35 per 100,000 from 1985 to 2010 and started to decline in the recent decade. Their tobacco consumption rates were very low in the 1950s, gradually increasing from 1955 to 1975, then declining slowly in the recent three decades. Without a clear timing or evidence of reductions in tobacco consumption, the best-estimated lag years between tobacco consumption and lung cancer incidence are 17 years for males and 31 years for females, a 14-year difference in lag times between men and women in Norway.

In Sweden, the LCIRs for males are at relatively low levels and did not change significantly over the past five decades, constantly increasing before 1980 and slowly declining in recent decades. This is consistent with the pattern of their tobacco consumption also at a lower level, somewhat increasing before the 1970s, then declining in recent decades. For Swedes, the best-estimated lag years between tobacco consumption and lung cancer incidence are 13 years for males and 32 years for females.

The results are in general agreement with the findings in the literature that there is a lag of 15–30 years at the population level between cigarette smoking and lung cancer mortality, and the lag time is somewhat longer for women [15,16,17,18,22,23]. The lag time between cigarette smoking and lung cancer incidence could be slightly shorter than the lag time between cigarette smoking and lung cancer mortality.

**Figure 4:**
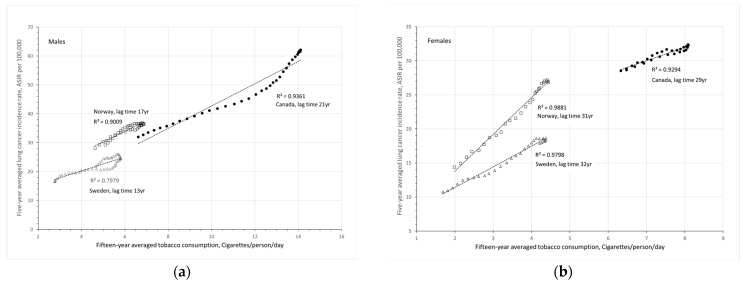
Five-year averaged lung cancer incidence rates as a function of 15-year averaged tobacco consumption with considerations of respective lag times for men and women in Canada, Norway and Sweden: (**a**) males and (**b**) females.

To consider the long-term effect of tobacco smoking and to limit the impact from various statistical uncertainties and fluctuations, 15-year averaged tobacco consumption rates (TC15) and 5-year averaged lung cancer incidence rates (LCIR5) are calculated. For example, annual LCIRs from 1989 to 1993 were averaged to represent the LCIR5 of 1991. For Canadian females with a lag time of 29 years, the LCIR5 of 1991 corresponds to the TC15 in 1962 (averaged from the values of 1955 to 1969 with 1962 at the center of the 15-year period). Moving-window averages are applied until the end of available data for recent years. Figure 4 presents 5-year averaged LCIRs as a function of 15-year averaged TC statistics with considerations of the respective lag times in Canada, Norway and Sweden for men and women, respectively.

The slopes of the trendlines in Figure 4 represent the increase in lung cancer incidence rate (ASIR per 100,000) for the increase of one cigarette per person per day after the respective lag times. These values are summarized in Table 1. On average, one more cigarette smoked per person per day can increase lung cancer cases by 3.4 ± 1.3 per 100,000 population.

## 4. Discussion

The results in Figure 4 clearly show that lung cancer incidence rates correlate very well with the tobacco consumption rates with *R*^2^≥0.80. Higher smoking rates resulted in higher lung cancer incidence rates decades later. In the early 1900s, lung cancer incidence rates increased proportionally with the increasing smoking rates in many countries around the world. With the introduction of tobacco control, even before the WHO Framework Convention on Tobacco Control [24] and proactive national programs [25,26,27], lung cancer incidence rates have been decreasing with decreasing smoking rates over the past decades. In Canadian men, the drop in smoking began in the mid-1960s, preceding the drop in lung cancer incidence by 21 years. In 2010, the lung cancer incidence rate for Canadian men was 38 per 100,000 reduced from 60 per 100,000 in 1990, which reflects the reduction in tobacco consumption from 14 cigarettes/person/day around 1969 to 9 cigarettes/person/day around 1989. A 36% reduction in tobacco smoking resulted in about 37% reduction in lung cancer cases among Canadian men.

In the last century, there were many more female smokers in Canada than in Nordic countries. For Canadian women, tobacco consumption began to drop in the mid-1980s, preceding the drop in lung cancer incidence by 29 years. The lung cancer incidence rate for Canadian women was 31 per 100,000 in 2010, a factor of 1.2 higher than the rate of 26 per 100,000 in Norway and a factor of 1.6 higher than the rate of 19 per 100,000 in Sweden. The difference in LCIRs reflects the difference in TC15 rates between women in Canada and Sweden—8.0 cigarettes/person/day around 1981 in Canada, a factor of 1.9 higher than the rate of 4.2 cigarettes/person/day around 1978 in Sweden. While the difference between LCIRs in Canada and Sweden can be reasonably explained, the difference between LCIRs in Norway (26 per 100,000 in 2010) and Sweden (19 per 100,000 in 2010) cannot be explained by the same 15-year averaged TC rate of 4.2 cigarettes/person/day in the 1970s and 1980s, as shown in the last column of Table 2.

For women, the tobacco consumption rates were very similar in Norway and Sweden before 1990. After 1990, the smoking rate in Norway fluctuated around 4.5 cigarettes/person/day while the rate in Sweden has continued to decline to below 3 cigarettes/person/day. Even though there is a long lag time of about 30 years for tobacco-induced lung cancer, more recent smoking activities may significantly contribute to the induction of lung cancer. To examine this, the 25-year averaged tobacco consumption rates, TC25 (i.e., tobacco consumption between 5 and 30 years before the year of incidence), are calculated. Since tobacco consumption rates are only available up to 2005, the calculations of TC25 (averaged from 1981 to 2005) are for LCIRs in 2010 (averaged from 2008 to 2012). The results are listed in Table 2. The results confirmed the chronic and accumulative effect of tobacco smoking on the induction of lung cancer. LCIR increases proportionally with the increase of TC25. The Canadian LCIR being 1.2 times higher than the rate in Norway and 1.6 times higher than the rate in Sweden can be reasonably well explained by the fact that the Canadian female TC25 rate is 1.3 times higher than the TC25 rate in Norway and 1.6 times higher than the TC25 rate in Sweden.

As demonstrated in the literature, this study also found that the lag time between the population’s average smoking rate and the incidence rate for all types of lung cancers may vary considerably across populations and countries. This is because many factors affect the lag time, such as changes or details in tobacco products, ages of smoking initiation and durations of smoking as well as histological types of lung cancer. Since the detailed information, most being data at the individual level, is not available publicly, this study is an ecological study based on national population average data.

## 5. Conclusions

At the population average level, historical data clearly demonstrated that age-standardized incidence rates for all types of lung cancers increase proportionally with increasing population average tobacco consumption rates, as measured via the number of cigarettes per person per day and that the impact of smoking affects the lung cancer incidence rate for decades even as smoking rates drop. The best fitting assumed a lag of 21 years for men and 29 years for women in Canada. Even at very similar socioeconomic status and similar social determinants of health and environmental factors, due to the fact that Canadian female tobacco consumption rates are, on average, 1.3 times higher than the rates in Norway and 1.6 times higher than the rates in Sweden averaged from 1981 to 2005, the Canadian female lung cancer incidence rate averaged from 2008 to 2012 is 1.2 times higher than the rate in Norway and 1.6 times higher than the rate in Sweden. On average, one more cigarette smoked per person per day can increase lung cancer cases by 2 to 5 per 100,000 population.

With strong tobacco control policies, fewer Canadians, as well as fewer Norwegians and Swedes, are smoking now, and they are also smoking less. In Canadian males, for example, the smoking rate dropped by 36% from 1969 to 1989, preceding a drop of 37% in lung cancer incidence by 21 years. While this is a positive development, the correlation between past smoking habits and lung cancer incidence is very strong and continues to contribute to the burden of lung cancer in Canada. The results could act as a reference point where preventive strategies like the WHO Framework Convention on Tobacco Control should be further reinforced in high HDI countries.

## Figures and Tables

**Table 1:** Increase in lung cancer incidence rate per 100,000 for the increase of one cigarette per person per day.

	Men	Women
Canada	3.85	1.96
Norway	3.66	5.40
Sweden	2.42	3.03
	3.31 ± 0.78	3.46 ± 1.76

**Table 2:** The 5-year averaged LCIRs around 2010, 25-year averaged TCs (1981–2005) and 15-year averaged tobacco consumption rates with lag year at the center of a 15-year range in Canada, Norway and Sweden.

Females	5-Year Averaged LCIR5 (2008–2012) ASIR per 100,000	25-Year Averaged TC25 (1981–2005) Cigarettes/Person/Day	15-Year Averaged TC15 Cigarettes/Person/Day
Canada	31	5.7	8.0 (1974–1988)
Norway	26	4.4	4.2 (1972–1986)
Sweden	19	3.5	4.2 (1971–1985)

## Data Availability

All data are available on public websites as given in the references.
